# Supplementary material for: The relationship between physical fitness and competitive performance of Taekwondo athletes
Source: PLoS One. 2022 Jun 24;17(6):e0267711. doi: 10.1371/journal.pone.0267711 (PMC9232157; doi:10.1371/journal.pone.0267711)

PLOS ONE

The relationship between physical fitness and competitive performance of Taekwondo athletes: An empirical research of the 2020 National Taekwondo Championship Series

--Manuscript Draft--

Manuscript Number:	PONE-D-21-18355
Article Type:	Research Article
Full Title:	The relationship between physical fitness and competitive performance of Taekwondo athletes: An empirical research of the 2020 National Taekwondo Championship Series
Short Title:	The relationship between physical fitness and competitive performance of Taekwondo athletes
Corresponding Author:	Lumin He<br>Beijing Sport University<br>Beijing, CHINA
Keywords:	Taekwondo; physical fitness; competition performance; 2020 National Taekwondo Championship Series
Abstract:	The competition and physical fitness test results of the 2020 National Taekwondo Championship Series were analyzed using curve fitting, linear regression, and other statistical methods. As far as we know, it is the first Taekwondo competition that uses physical fitness test (PFT) scores as the 8-in-4 selection criteria. The results show that the probability of the final total score of the series of championships entering the top 8 or top 3 is exponentially related to PFT results. It finds that athletes with better PFT scores are more likely to enter the quarterfinals. Among athletes entering the semifinals, the athlete with the best physical fitness has the greatest probability of winning the championship. The difference in physical fitness between athletes is mainly reflected in the 30-meter sprint. Overall, the competitive performance of professional Taekwondo athletes is significantly positively correlated with their physical fitness, especially for female Taekwondo athletes. Through the results obtained, it is concluded that Taekwondo athletes need to strengthen physical training, specifically in increase of explosive power.
Order of Authors:	Rui Liu
	Lumin He
Additional Information:	
Question	Response
**Financial Disclosure**<br><br>Enter a financial disclosure statement that describes the sources of funding for the work included in this submission. Review the submission guidelines for detailed requirements. View published research articles from *PLOS ONE* for specific examples.<br><br>This statement is required for submission and **will appear in the published article** if the submission is accepted. Please make sure it is accurate.	This study was supported by grant 201915002 from the Ocean University of China (Rui Liu received), and the grant “Elite coaches double hundred training plan implementation measures” from the General Administration of Sport of China. (Lumin He reveived).<br>The funders had no role in study design, data collection and analysis, decision to publish, or preparation of the manuscript.

Powered by Editorial Manager® and ProduXion Manager® from Aries Systems Corporation

**Unfunded studies**<br>Enter: *The author(s) received no specific funding for this work.*<br><br>**Funded studies**<br>Enter a statement with the following details:<br>• Initials of the authors who received each award<br>• Grant numbers awarded to each author<br>• The full name of each funder<br>• URL of each funder website<br>• Did the sponsors or funders play any role in the study design, data collection and analysis, decision to publish, or preparation of the manuscript?<br>• **NO** - Include this sentence at the end of your statement: *The funders had no role in study design, data collection and analysis, decision to publish, or preparation of the manuscript.*<br>• **YES** - Specify the role(s) played.<br><br>* typeset	
**Competing Interests**<br><br>Use the instructions below to enter a competing interest statement for this submission. On behalf of all authors, disclose any competing interests that could be perceived to bias this work—acknowledging all financial support and any other relevant financial or non-financial competing interests.<br><br>This statement is required for submission and **will appear in the published article** if the submission is accepted. Please make sure it is accurate and that any funding sources listed in your Funding Information later in the submission form are also declared in your Financial Disclosure statement.<br><br>View published research articles from *PLOS ONE* for specific examples.	The authors have declared that no competing interests exist.

**NO authors have competing interests**<br><br>Enter: *The authors have declared that no competing interests exist.*<br><br>**Authors with competing interests**<br><br>Enter competing interest details beginning with this statement:<br><br>*I have read the journal's policy and the authors of this manuscript have the following competing interests: [insert competing interests here]*<br><br>* typeset	
**Ethics Statement**<br><br>Enter an ethics statement for this submission. This statement is required if the study involved:<br><br>• Human participants<br>• Human specimens or tissue<br>• Vertebrate animals or cephalopods<br>• Vertebrate embryos or tissues<br>• Field research<br><br>Write "N/A" if the submission does not require an ethics statement.<br><br>General guidance is provided below. Consult the submission guidelines for detailed instructions. **Make sure that all information entered here is included in the Methods section of the manuscript.**	N/A

**Format for specific study types**<br><br>**Human Subject Research (involving human participants and/or tissue)**<br>• Give the name of the institutional review board or ethics committee that approved the study<br>• Include the approval number and/or a statement indicating approval of this research<br>• Indicate the form of consent obtained (written/oral) or the reason that consent was not obtained (e.g. the data were analyzed anonymously)<br><br>**Animal Research (involving vertebrate animals, embryos or tissues)**<br>• Provide the name of the Institutional Animal Care and Use Committee (IACUC) or other relevant ethics board that reviewed the study protocol, and indicate whether they approved this research or granted a formal waiver of ethical approval<br>• Include an approval number if one was obtained<br>• If the study involved *non-human primates*, add *additional details* about animal welfare and steps taken to ameliorate suffering<br>• If anesthesia, euthanasia, or any kind of animal sacrifice is part of the study, include briefly which substances and/or methods were applied<br><br>**Field Research**<br><br>Include the following details if this study involves the collection of plant, animal, or other materials from a natural setting:<br>• Field permit number<br>• Name of the institution or relevant body that granted permission	
**Data Availability**<br><br>Authors are required to make all data underlying the findings described fully available, without restriction, and from the time of publication. PLOS allows rare exceptions to address legal and ethical concerns. See the PLOS Data Policy and FAQ for detailed information.	Yes - all data are fully available without restriction

Powered by Editorial Manager® and ProduXion Manager® from Aries Systems Corporation

A Data Availability Statement describing where the data can be found is required at submission. Your answers to this question constitute the Data Availability Statement and **will be published in the article**, if accepted.<br><br>**Important:** Stating 'data available on request from the author' is not sufficient. If your data are only available upon request, select 'No' for the first question and explain your exceptional situation in the text box.<br><br>Do the authors confirm that all data underlying the findings described in their manuscript are fully available without restriction?	
**Describe where the data may be found in full sentences. If you are copying our sample text, replace any instances of XXX with the appropriate details.**<br><br>• If the data are **held or will be held in a public repository**, include URLs, accession numbers or DOIs. If this information will only be available after acceptance, indicate this by ticking the box below. For example: *All XXX files are available from the XXX database (accession number(s) XXX, XXX.)*.<br>• If the data are all contained **within the manuscript and/or Supporting Information files**, enter the following: *All relevant data are within the manuscript and its Supporting Information files.*<br>• If neither of these applies but you are able to provide **details of access elsewhere**, with or without limitations, please do so. For example:<br><br>*Data cannot be shared publicly because of [XXX]. Data are available from the XXX Institutional Data Access / Ethics Committee (contact via XXX) for researchers who meet the criteria for access to confidential data.*<br><br>*The data underlying the results presented in the study are available from (include the name of the third party*	All the data used in our paper can be found on the website of Chinese Taekwondo Association (http://www.taekwondo.org.cn/), however, the data in pdf files are write in Chinese, if anyone need it in English, feel free to contact the corresponding author.

Powered by Editorial Manager® and ProduXion Manager® from Aries Systems Corporation

and contact information or URL).<br>• This text is appropriate if the data are owned by a third party and authors do not have permission to share the data.<br><br>* typeset	
Additional data availability information:	

Powered by Editorial Manager® and ProduXion Manager® from Aries Systems Corporation

manuscript Click here to access/download;Manuscript;manuscript.docx

The relationship between physical fitness and competitive performance of Taekwondo athletes

Rui Liu[1], Lumin He[2*]

[1]Department of Physical Education, Ocean University of China, Qingdao 266100, China

[2]Affiliated Athletics School of Beijing Sport University, Beijing 100084, China

[*]Corresponding author:

E-mail: helumin@bsu.edu.cn

Abstract

The competition and physical fitness test results of the 2020 National Taekwondo Championship Series were analyzed using curve fitting, linear regression, and other statistical methods. As far as we know, it is the first Taekwondo competition that uses physical fitness test (PFT) scores as the 8-in-4 selection criteria. The results show that the probability of the final total score of the series of championships entering the top 8 or top 3 is exponentially related to PFT results. It finds that athletes with better PFT scores are more likely to enter the quarterfinals. Among athletes entering the semifinals, the athlete with the best physical fitness has the greatest probability of winning the championship. The difference in physical fitness between athletes is mainly reflected in the 30-meter sprint. Overall, the competitive performance of professional Taekwondo athletes is significantly positively correlated with their physical fitness, especially for female Taekwondo athletes. Through the results obtained, it is concluded that Taekwondo athletes need to strengthen physical training, specifically in increase of explosive power.

1. Introduction

Taekwondo originated in the Korean peninsula, which is famous for its flexible kicking techniques, and has been an Olympic combat discipline since 2000. In order to guide the training and competition more scientifically, reasons affecting the achievements of Taekwondo athletes have been studied extensively during the past few

decades. In general, performance in taekwondo is considered to be determined by a competitor’s technical, tactical, psychological, physical and physiological characteristics [1, 2].

Since the establishment of the World Taekwondo Federation (WTF) in 1973, the competition rules of Taekwondo have been revised more than 20 times. The changes in the rules have also led to gradual increase in the antagonism between two competitors. In competitions conducted according to the previous rules, Taekwondo athletes did not generally apply difficult technical approaches, but preferred to use secure frontal skills to win the game, which is why the roundhouse kick was the most popular means of scoring [3]. However, following the changes in competition rules, Taekwondo competition tactics now have offensive tactics as the main approach and counter-attack tactics as the secondary approach [4]. As an important foundation of athletes' competitive ability, physical fitness has also become increasingly prominent with the rule changes [5].

In terms of physiological characteristics, it has been found that elite taekwondo athletes tend to possess low levels of body fat [6, 7], moderate to high levels of cardio-respiratory fitness [8] and high levels of both aerobic and anaerobic physical fitness [9], while muscle strength is often not a key role [10]. In terms of specific physical fitness characteristics, successful taekwondo athletes were found to possess significantly higher maximum running speed [6], better performance on 30m run, counter movement jump, moving sideways and walking backwards [11]. Additionally, research on the physical fitness test (PFT) of junior taekwondo athletes revealed that the power of lower

extremities, strength and endurance are of great importance to the sports result [12, 13]. However, the aforementioned studies are limited by the fact that the PFTs performed are all conducted during training, because there is a big difference between training and competition modes for athletes' psychological and physical conditions [9, 14, 15]. Therefore, further study on the relationship between PFT and athletes' competition performance is still required.

Aware of the important role of physical fitness for athletes' competitive performance, the General Administration of Sport of China issued the "Notice on Further Strengthening Basic Physical Training and Complementary Physical Ability" (hereinafter "the Notice") on February 24, 2020, which stipulates the competition system of combining physical fitness test with competition results. After that, the 2020 National Taekwondo Championship Series (NTCS) held in Wuxi, Jiangsu Province became the first taekwondo competition with PFT added to the rules, which also provided valuable data for the study of the relationship between athletes' competition performance and PFT.

The main aim of this paper is to study the relationship between PFT results and taekwondo athletes' competitive performance by analyzing the results of four races in the 2020 NTCS. As a supplement to the research on physiological indicators and PFT in training, this research hopes to use empirical research to provide a more macroscopic view of the role of physical fitness in Taekwondo competitions.

2. Materials and methods

The first and second races of 2020 NTCS were held from September 22nd to 25th

and September 27th to 30th, respectively. Prior to this, the first PFT (PFT1) was conducted from September 19th to 20th. The second PFT (PFT2), the third race and the championship final (hereafter referred as the fourth races) were held during October 15th to October 28th. The competition rules adopted for the NTCS is "World Taekwondo Federation Para Taekwondo Competition Rules" (in force as of May 15, 2019) [16]. A three-round total points system was carried out, each round is 2 minutes, and there is a 1-minute break between rounds. There was a total of 638 professional Taekwondo athletes (393 males and 245 females) who participated; the number of athletes in each race may vary slightly due to injuries or other reasons.

There are 5 items in the PFT, namely: weight-bearing squat, abdominal muscle endurance, back muscle endurance, 30-meter run and 3000-meter run (full marks of each item are the same). The grading standard is given in **Table 1**. The evaluation standard of squat strength is the ratio of the maximum squat weight to the body weight. When testing the endurance of the abdominal muscles, the athletes lie on their back on the jumping box, keeping their body on a flat surface. The test method of back muscle endurance is the same as that of abdominal muscle endurance, except that the athlete lies on the jumping box.

Table 1. **Assessment matrix for the physical fitness test**

Score	Weight-bearing squat (Multiples of	30-meter run	Back muscle endurance	Abdominal muscle endurance	3000-meter run (minute:second)	
					Male	Female

	weight)	(s)	(s)	(s)		
20	≥1.2	≤4.5	≥120	≥120	≤11:00	≤11:30
19	1.18	4.55	118-119	118-119	11:01-11:10	11:31-11:40
18	1.16	4.6	116-117	116-117	11:11-11:20	11:41-11:50
17	1.14	4.65	114-115	114-115	11:21-11:25	11:51-11:55
16	1.12	4.7	112-113	112-113	11:26-11:30	11:56-12:00
15	1.1	4.75	110-111	110-111	11:31-11:35	12:01-12:05
14	1.08	4.8	108-109	108-109	11:36-11:40	12:06-12:10
13	1.06	4.85	106-107	106-107	11:41-11:45	12:11-12:15
12	1.04	4.9	104-105	104-105	11:46-11:50	12:16-12:20
11	1.02	4.95	102-103	102-103	11:51-11:55	12:21-12:25
10	1.0	5.0	100-101	100-101	11:56-12:00	12:26-12:30
9	0.95	5.05	95-99	95-99	12:01-12:10	12:31-12:40
8	0.9	5.1	90-94	90-94	12:11-12:20	12:41-12:50
7	0.85	5.15	85-89	85-89	12:21-12:30	12:51-13:00
6	0.8	5.2	80-84	80-84	12:31-12:45	13:01-13:15
5	0.75	5.25	75-79	75-79	12:46-13:00	13:16-13:30
4	0.7	5.3	70-74	70-74	13:01-13:15	13:31-13:45
3	0.65	5.35	65-69	65-69	13:16-13:30	13:46-14:00
2	0.6	5.4	60-64	60-64	13:31-13:45	14:01-14:15

1	0.55	5.45	30-59	30-59	13:46-14:00	14:16-14:30
0	≤0.5	＞5.5	＜30	＜30	＞14:00	＞14:30

The ranking and points of the four races and the scores of two PFTs are used in this research. It is worth noting that after the top 8 players are produced, the top four players in PFT directly enter the semi-finals, and the last 4 players in the PFT are regarded as tied for fifth.

Data statistics, fitting and linear regression analysis were all performed with MATLAB software version R2021a. All p-values in this article are less than 0.001, and will not be marked hereafter.

3. Results and discussion

3.1 Correlation between competition results and PFT results

3.1.1 Total points

We consider first the correlation between the competition final points and the PFT results. The scoring rule of the NTCS listed in **Table 2** can be calculated with the following formula: $S=\left[10\times 166.7e^{-0.5108\times M}\right]/10$, where S represents points, M is determined by the competition ranking and bracket means rounding function. Since the number of athletes in each heavyweight is different, the percentage of PFT ranking is used in this research regarding to it's more meaningful to facilitate the comparison among different heavyweights.

Table 2. Scoring rule of the 2020 NTCS

Rank	No. 1 (M=1)	No. 2 (M=2)	Joint third (M=3)	Joint fifth (M=4)
Point	100	60	36	21.6

Fig 1 displays the proportion of athletes with different PFT ranking percentages among the athletes who have entered the top 8 and top 3 in the final points. The proportions were fitted to the results of PFT1, PFT2 and the average of these two ($\overline{\text{PFT}}$). It can be found that the proportions can be well fitted by exponential function with the shape of $y = ae^{bx}$, where a and b are constant coefficients. The coefficients calculated by the least squares method, the Goodness of Fit (R^2) and the number of athletes (N) are listed in **Table 3** and **Table 4**. For all athletes, female athletes and male athletes entering the top 8 (Fig. 1a-c), the R^2 are very encouraging and all above 0.8, although with a slight decline when fitted with PFT2 results. Potential possible causes for this phenomenon will be discussed later. The fitting in entering the top 3 seems even better, except for the fitting with the $\overline{\text{PFT}}$ of male athletes, which has the minimum R^2 value (0.788). Compared with **Fig 1a-c**, the fitting curves in **Fig 1d-f** are steeper, which means that athletes tend to have a smaller probability to enter the top 3 in the final total points with the lower PFT ranking.

Fig 1. The percentage of all athletes (top row), female athletes (middle row) and male athletes (bottom row) in each PFT ranking segment among the top 8 (a-c) and top 3 (d-f) players in the final total points. The blue, red and green solid lines

correspond to the fitting curves of PFT1, PFT2 and $\overline{\mathrm{PFT}}$, respectively.

Table 3. The fitting coefficient, the Goodness of Fit and the number of athletes in Fig. 1a-c.

	All			Female			Male		
	PFT1	PFT2	$\overline{\mathrm{PFT}}$	PFT1	PFT2	$\overline{\mathrm{PFT}}$	PFT1	PFT2	$\overline{\mathrm{PFT}}$
a	0.305 ± 0.037	0.276 ± 0.067	0.287 ± 0.043	0.289 ± 0.072	0.265 ± 0.081	0.264 ± 0.068	0.326 ± 0.079	0.288 ± 0.094	0.315 ± 0.043
b	-2.861 ± 0.505	-2.49 ± 0.913	-2.626 ± 0.579	-2.641 ± 0.978	-2.354 ± 1.097	-2.346 ± 0.926	-3.149 ± 1.093	-2.649 ± 1.28	-2.956 ± 0.591
R^2	0.974	0.89	0.959	0.893	0.834	0.873	0.907	0.827	0.968
N	573	520	638	219	206	245	354	314	393

Table 4. Same as Table 3 but for Fig. 1d-e.

	All			Female			Male		
	PFT1	PFT2	$\overline{\mathrm{PFT}}$	PFT1	PFT2	$\overline{\mathrm{PFT}}$	PFT1	PFT2	$\overline{\mathrm{PFT}}$
a	0.602 ± 0.054	0.497 ± 0.135	0.57 ± 0.129	0.63 ± 0.151	0.434 ± 0.12	0.581 ± 0.149	0.573 ± 0.102	0.582 ± 0.243	0.567 ± 0.295
b	-5.811 ± 0.719	-4.635 ± 1.753	-5.71 ± 1.771	-5.975 ± 1.958	-3.898 ± 1.52	-6.104 ± 2.137	-5.641 ± 1.386	-5.6 ± 3.218	-5.453 ± 3.899
R^2	0.992	0.925	0.951	0.945	0.916	0.938	0.969	0.854	0.788

Moreover, it is obvious that the exponential function can fit better than the linear

function according to the distribution of data. This may be due to the fact that the PFT rank is the only basis for eight-to-four promotion, which means after entering the quarterfinals, the top 4 athletes in PFT will inevitably get more points than the bottom 4 ones. On the other hand, the exponential scoring rule also plays an important role in widening the points gap between athletes.

3.1.2 Entering the quarterfinals

As aforementioned, athletes who enter the semifinals are screened by PFT rankings. Thus, in order to study the relationship between athletes' physical fitness and competition performance, the impact of the rules must be excluded. In this section, the statistics are made on the PFT results of the top 8 athletes in each race.

Fig 2 illustrates that, in general, the top 10% of athletes in PFT ranking account for the highest proportion of the top 8 players in the four races. As the PFT ranks lower, this proportion is also lowers. This feature is more obvious for the statistics of all athletes (**Fig 2a-d**) than for females (**Fig 2e-h**) and males (**Fig 2i-l**) specifically. In addition, the proportion of athletes in the top 50% and bottom 50% of the PFT was also counted. Apparently, the top 50% account for a higher proportion of the top 8 players (about 2/3). For female Taekwondo athletes, the top 50% in the PFT accounted for a higher proportion in the top 8, implying that compared with male athletes, physical fitness is more crucial in competition performance for female athletes. A linear correlation analysis was performed on the proportions of athletes with different PFT ranking percentages among all the top 8 players (**Fig 3**), all coefficients are calculated within the 95% confidence interval and p value less than 0.001. The high correlation

coefficient (r=0.914) illustrates that there is a significantly negative correlation between the PFT ranking percentage and its proportion in the top 8 athletes. It is noteworthy that the sample size of athletes in this study is 638, so the results are considered statistically significant.

Fig 2. The percentage of all athletes (a-d), female athletes (e-h) and male athletes (i-l) ranked by PFT among the top 8 athletes in the four races, each row in this figure is the results of one race.

Fig 3. Linear regression of the proportion of athletes in each PFT ranking interval among the top 8 athletes. The regression equation and correlation coefficient are also listed.

3.1.3 Winning in semifinals

This section will discuss whether athletes with better physical fitness are more likely to win among the 4 players who have entered the semifinals. **Table 5** counted the frequencies of the athletes ranked first, second, third and fourth in PFT who won the championship after entering the semifinals. Since both female and male competitors are divided into eight heavyweights, the number '4' in the first row and first column means that in the 8 semifinals of the first race, 4 of the athletes who ranked first in the PFT won the championship. Obviously, assuming that every athlete who enters the semifinals has an equal chance of winning the championship, the expectation of this value is 2.

Table 5. Frequency statistics of top 4 athletes with different PFT rankings winning the chanmpionship.

PFT rank	No. 1		No. 2		No. 3		No. 4	
Race	Female	Male	Female	Male	Female	Male	Female	Male
1	4	3	1	3	2	2	1	0
2	3	3	3	2	1	1	1	2
3	4	3	2	2	1	2	1	1
4	5	3	0	4	2	1	1	0
Average	4	3	1.5	2.75	1.5	1.5	1	0.75

For females, the average number of times that the athletes with the first PFT won the championship is 4, which is twice the expected value. The second and the third athletes in the PFT have the same frequency of winning the championship, both are 1.5, which is slightly less than the expected value. The athletes ranked last in the PFT have the least frequency of winning. The same is true for male athletes, but the difference in the frequency of winning championship between the first and the second in the PFT is not as large as that of female athletes. However, the above results all confirm that PFT results are indeed related to athletes' competitive performance, that is, athletes with better physical fitness have a greater probability of having better competitive performance. Furthermore, for female athletes entering the semifinals, the advantage brought by physical fitness may be even greater [12].

3.2 Key physical indicators

The scores of the athletes' 5 individual PFT items are respectively counted (**Fig 4**). As is illustrated, almost all players scored 19-20 points in weight-bearing squat, abdominal muscle endurance and back muscle endurance. This means that the squat strength, abdominal muscle endurance and back muscle endurance of these professional Taekwondo athletes are pretty good. The gap between individual athletes' PFT results comes from the 30-meter sprint. Female athletes generally didn't score high in the 30-meter sprint (**Fig 4d**), most athletes scored less than 10 points. Nevertheless, compared with other athletes, the proportion of female athletes who entered the top 8 in the competition has higher scores than other athletes. Male athletes score relatively higher than female athletes in the 30-meter sprint (**Fig 4i**), but similarly, top 8 athletes score higher than other athletes. Male athletes also generally perform better than female athletes in the 3000-meter run, and compared with other athletes, the percentage of top 8 athletes getting high scores is only slightly larger. For female athletes, the top 8 athletes also have a slightly better 3000m performance than other athletes, but not by too much.

Fig. 4. The proportion of top 8 athletes and other athletes in each score segment of each individual PFT item. The left column is for female athletes, and the right column is for male athletes.

Therefore, these statistical results demonstrate that the physical fitness gap of

Taekwondo athletes mainly depends on explosive power. Excellent athletes (corresponding to the top 8 athletes in this study) have better explosive power than other athletes [6, 17, 18]. For professional athletes, there is not much difference in leg strength, abdominal muscles, back muscle endurance and aerobic capacity.

3.3 PFT total scores

The average and standard deviation of PFT total scores of different heavyweight athletes are counted in this section. **Fig 5** shows that the PFT scores of female athletes are generally lower than that of male athletes, although the PFT standards of female and male are different. For both female and male athletes, the higher heavyweight athletes tend to obtain lower PFT scores. According to results mentioned in the previous section, it may be due to heavier athletes typically having relatively weaker explosive power.

Fig 5. PFT scores of athletes in different heavyweight classes, mean value and standard deviation are represented by box and solid line, respectively. Scores of the national team athletes are marked by five-pointed stars.

Another interesting thing is that for almost every heavyweight class, the average score of the PFT2 is better than that of the PFT1. This may explain the results in section 3.1.1. That is, after the PFT1, the first and the second races, the athletes realized that their physical deficiencies affected their performance in the competition, so they trained to improve their physical fitness. And with its improvement, the physical fitness gap

between competitors has less impact on the competition, that’s considered to be a reasonable explanation for the slight decline in R^2 when fitting with PFT2 ranking percentages.

In addition, thanks to the support of the Chinese National Taekwondo Team, several Taekwondo players who had won Olympic or World Championships also participated in the second PFT2, and their PFT scores are also marked in **Fig 5**. Not surprisingly, these few athletes who had won world championships have quite good physical test results. Their scores are all higher than the average, especially the scores of Jingyu Wu, Lijun Zhou and Shuai Zhao which exceeds the average plus one standard deviation. This result also provides a strong evidence that physical fitness is a necessary condition for excellent Taekwondo athletes.

4. Conclusions

This study conducted a statistical analysis of the competition results and PFT results of the 2020 NTCS, which is the first Taekwondo major event after the General Administration of Sport of China issued the Notice and provided a relatively large data set. Overall, the results demonstrate there is a significant positive correlation between athletes’ competition performance and PFT ranking.

As athletes rank lower in PFT, their proportion of the top 8 players in the final total points decreases exponentially, and the proportion decline even faster of the top 3. In each race, the top 50% in PFT account for more in the athletes entered quarterfinal. And in the semi-finals, athletes who are also ranked first in the PFT have a greater chance of coming to win the championship. The above three results all imply that the difference

in physical fitness does play a key role in the athletes' competition performance. For female athletes, the difference in physical fitness is relatively large, and there is also a large room for improvement in physical fitness; this conclusion is consistent with the conclusions in other related studies [6, 12]. Therefore, we can easily recommend that female athletes should pay more attention to improving their physical fitness. Especially after entering the semifinals, due to intensifying competition, the advantage of physical fitness will be more obvious. Furthermore, the key to the difference of Taekwondo Athletes' physical fitness lies in their explosive power, which is reflected in the biggest difference in the score of 30 meter sprint. This is also consistent with the characteristics that taekwondo is an intermittent high-intensity sport.

Finally, different from other related studies, due to the PFT in this study is related to the promotion, the athletes attached great importance to it, which makes this research more meaningful. In addition, this study is a supplement to research on physiological indicators, as it analyses and provides a macroscopic view of the role of physical fitness in Taekwondo competitions.

Acknowledgments

The authors wish to thank all the athletes participated in the 2020 National Taekwondo Championship Series.

References

1. Pieter W. Performance characteristics of elite taekwondo athletes. Korean J Sport Sci. 1991;3(1): 94-117.

2. Pieter W, Mateo C, Bercades LT. Determinants of Performance in Taekwondo. Med Sci Sport Exerc. 2002;34(5): 65.

3. Kang X, Liu Y. Technology of Taekwondo Athletes Application and The Winning Factor Analysis. Sichuan Sports Sci. 2015; 5.

4. Xiangjun L. Score Skills of the 16th Asian Games of Taekwondo Competition in New Rules. J Shenyang Sport Uni. 2012; 2.

5. Janowski M, Zieliński J, Kusy K. Exercise Response to Real Combat in Elite Taekwondo Athletes Before and After Competition Rule Changes. J Strength Cond Res. 2019.

6. Marković G, Mišigoj-Duraković M, Trninić S. Fitness profile of elite croatian female taekwondo athletes. Coll Antropol. 2005;29(1): 93-99.

7. Kazemi M, Waalen J, Morgan C, White AR. A profile of Olympic Taekwondo competitors. J Sport Sci Med. 2006;5(CSSI-1): 114.

8. Bridge CA, Ferreira Da Silva Santos J, Chaabène H, Pieter W, Franchini E. Physical and physiological profiles of taekwondo athletes[J]. Sports Medicine. 2014;44(6): 713-733.

9. Bouhlel E, Jouini A, Gmada N, Nefzi A, Ben Abdallah K, Tabka Z. Heart rate and blood lactate responses during Taekwondo training and competition. Sci Sport. 2006;21(5): 285-290.

10. Kim H-B, Jung H-C, Song J-K, Chai J-H, Lee E-J. A follow-up study on the physique, body composition, physical fitness, and isokinetic strength of female collegiate Taekwondo athletes. J Exerc Rehabil. 2015;11(1): 57.

11. Wazir MRWN, Hiel M Van, Mostaert M, Deconinck FJA, Pion J, Lenoir M. Identification of elite performance characteristics in a small sample of taekwondo athletes. PLoS One. 2019;14(5): e0217358.

12. Miller J, Bujak Z, Miller M. Sports result vs. general physical fitness level of junior taekwondo athletes. J Combat Sport Martial Arts. 2011;2(1): 39-44.

13. Sadowski J, Gierczuk D, Miller J, Cieśliński I, Buszta M. Success factors in male WTF taekwondo juniors. J Combat Sport Martial Arts. 2012;3(1): 47-51.

14. Bridge CA, McNaughton LR, Close GL, Drust B. Taekwondo exercise protocols do not recreate the physiological responses of championship combat. Int J Sports Med. 2013;34(7): 573-581.

15. Maloney MA, Renshaw I, Headrick J, Martin DT, Farrow D. Taekwondo fighting in training does not simulate the affective and cognitive demands of competition: Implications for behavior and transfer. Front Psychol. 2018;9(JAN): 25.

16. World Taekwondo. Competition rules a Interpretation. Opera [Internet]. 2019;56. Available from: http://www.worldtaekwondo.org/viewer_pdf/external/pdfjs-2.1.266-dist/web/viewer.html?file=http://www.worldtaekwondo.org/wp-content/uploads/2019/08/WT-Competition-Rules-Interpretation-Manchester-May-15-2019.pdf

17. Cular D, Krstulović S, Katić R, Primorac D, Vucić D. Predictors of fitness status on

success in Taekwondo. Coll Antropol. 2013;37(4): 1267-1274.

18. Monks L, Seo MW, Kim HB, Jung HC, Song JK. High-intensity interval training and athletic performance in Taekwondo athletes. J Sports Med Phys Fitness. 2017;57(10): 1252-1260.

Figure1

Click here to access/download;Figure;Fig1.tif

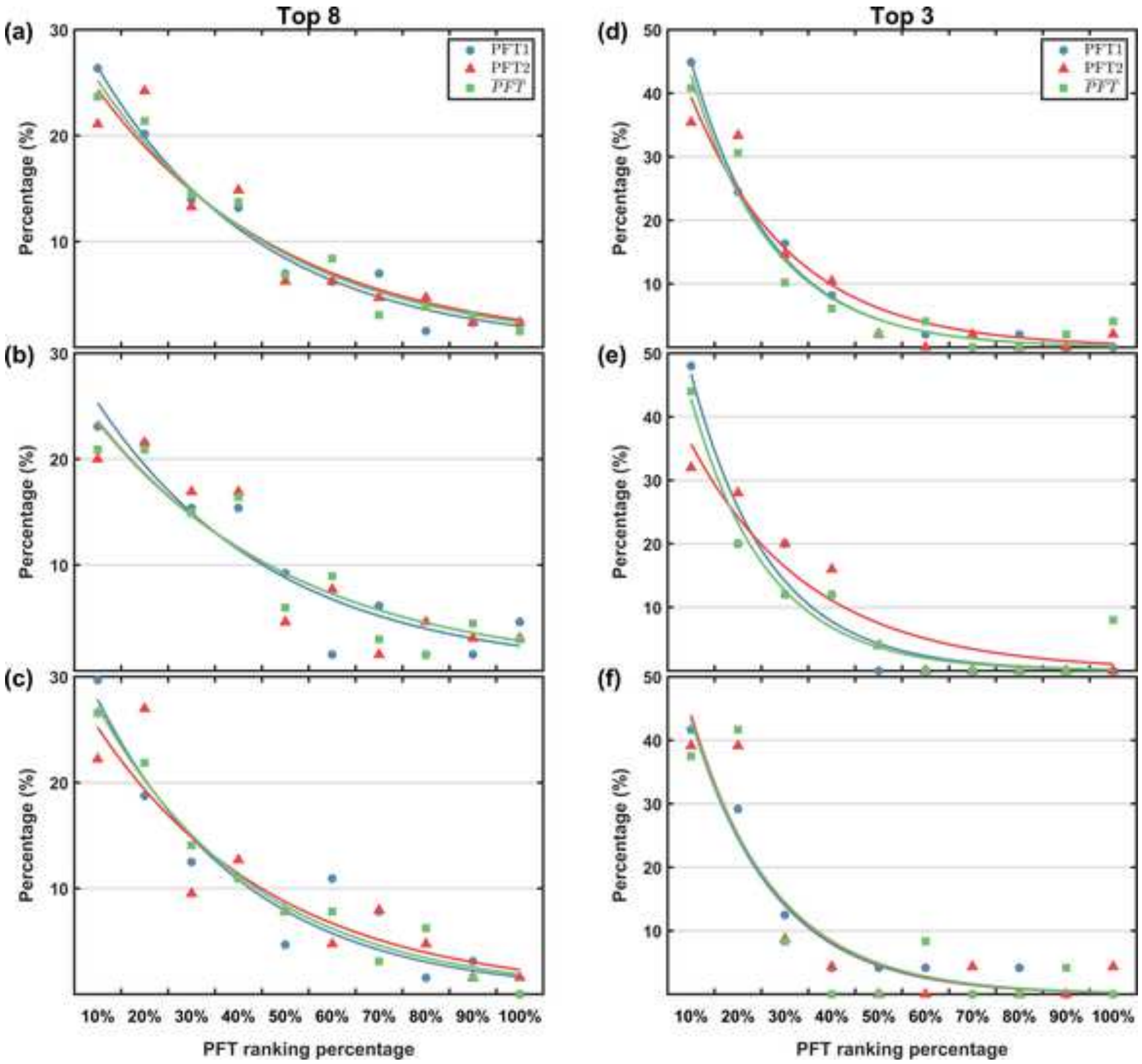

Figure2

Click here to access/download;Figure;Fig2.tif

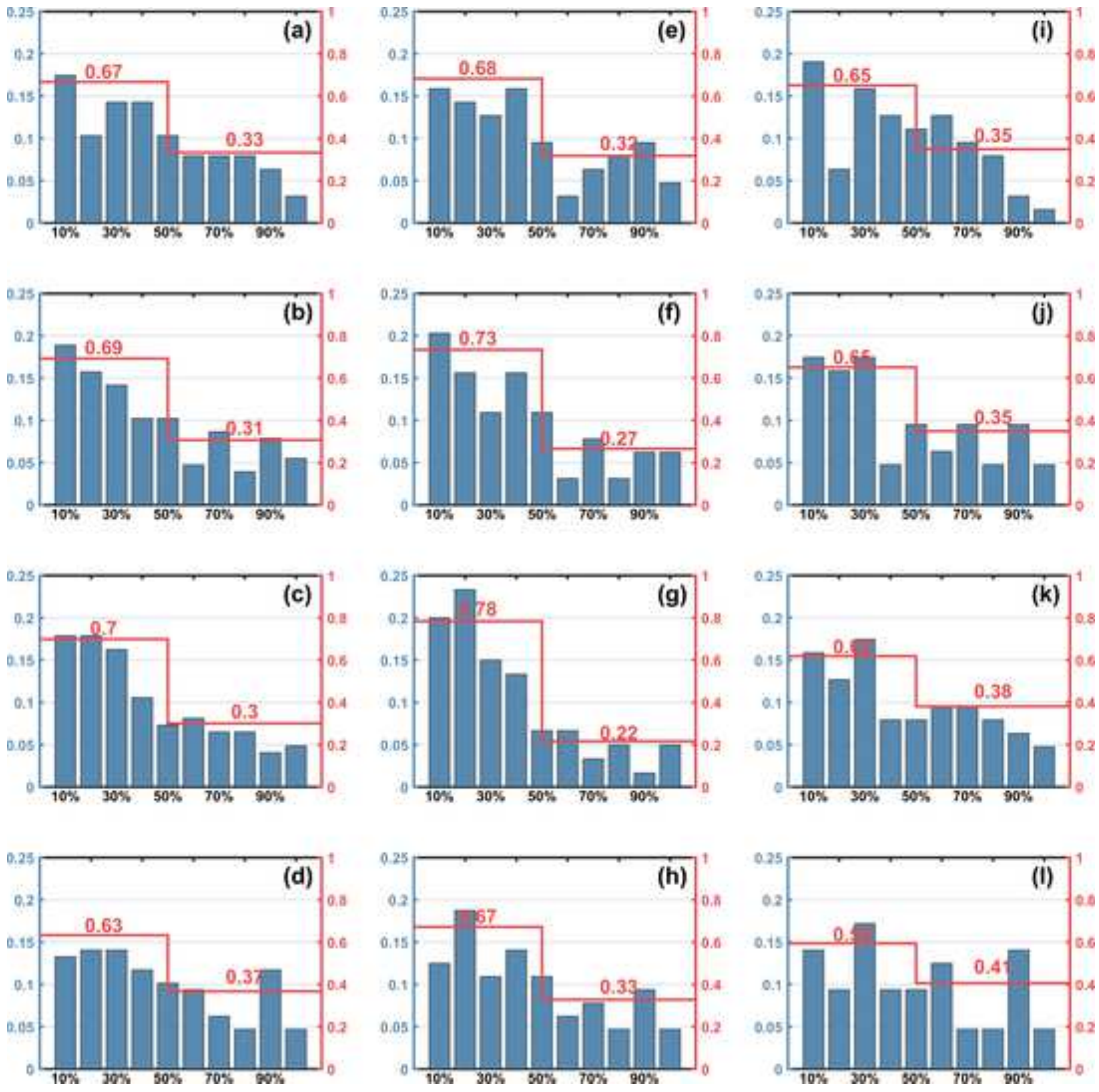

Figure3

Click here to access/download;Figure;Fig3.tif

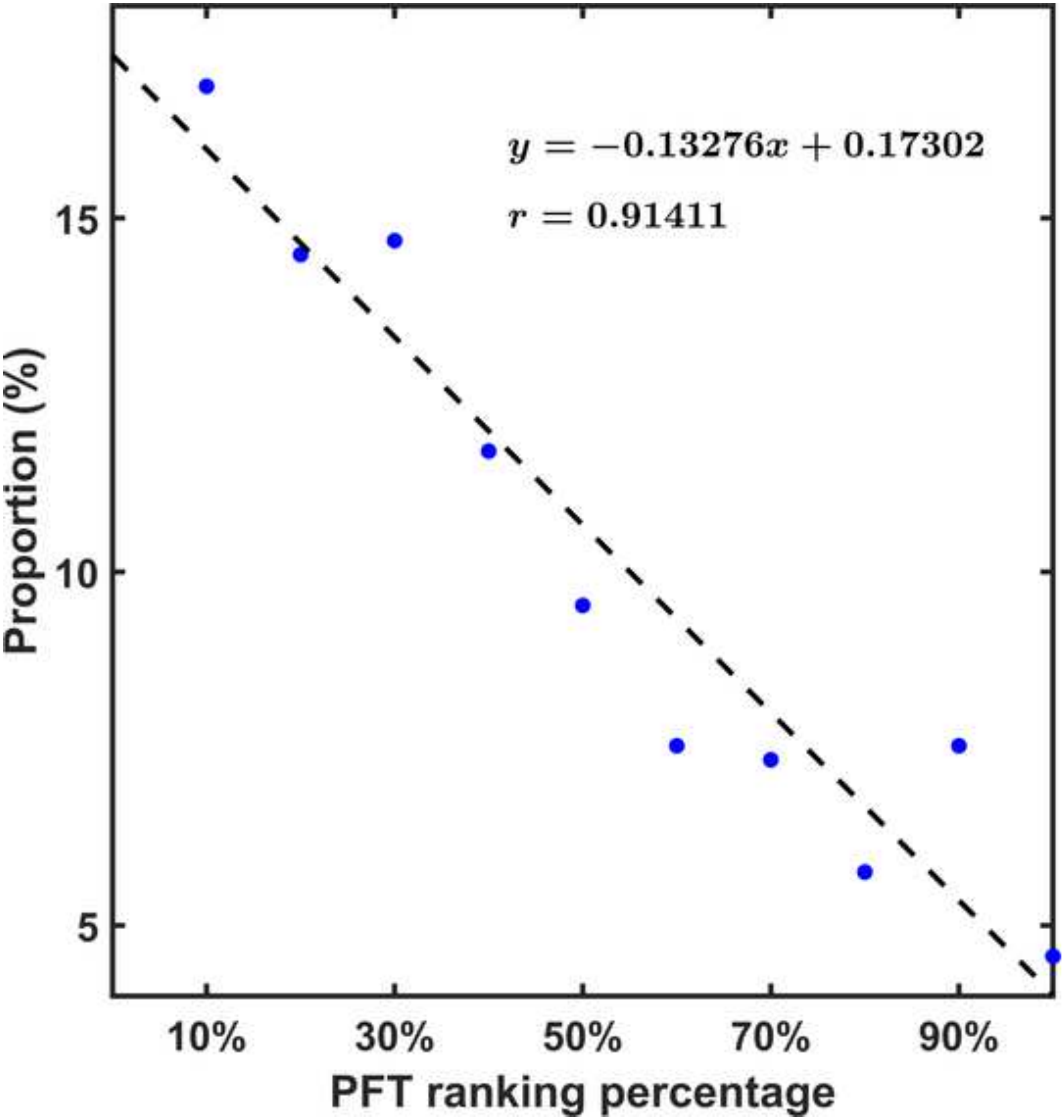

Figure4 Click here to access/download;Figure;Fig4.tif

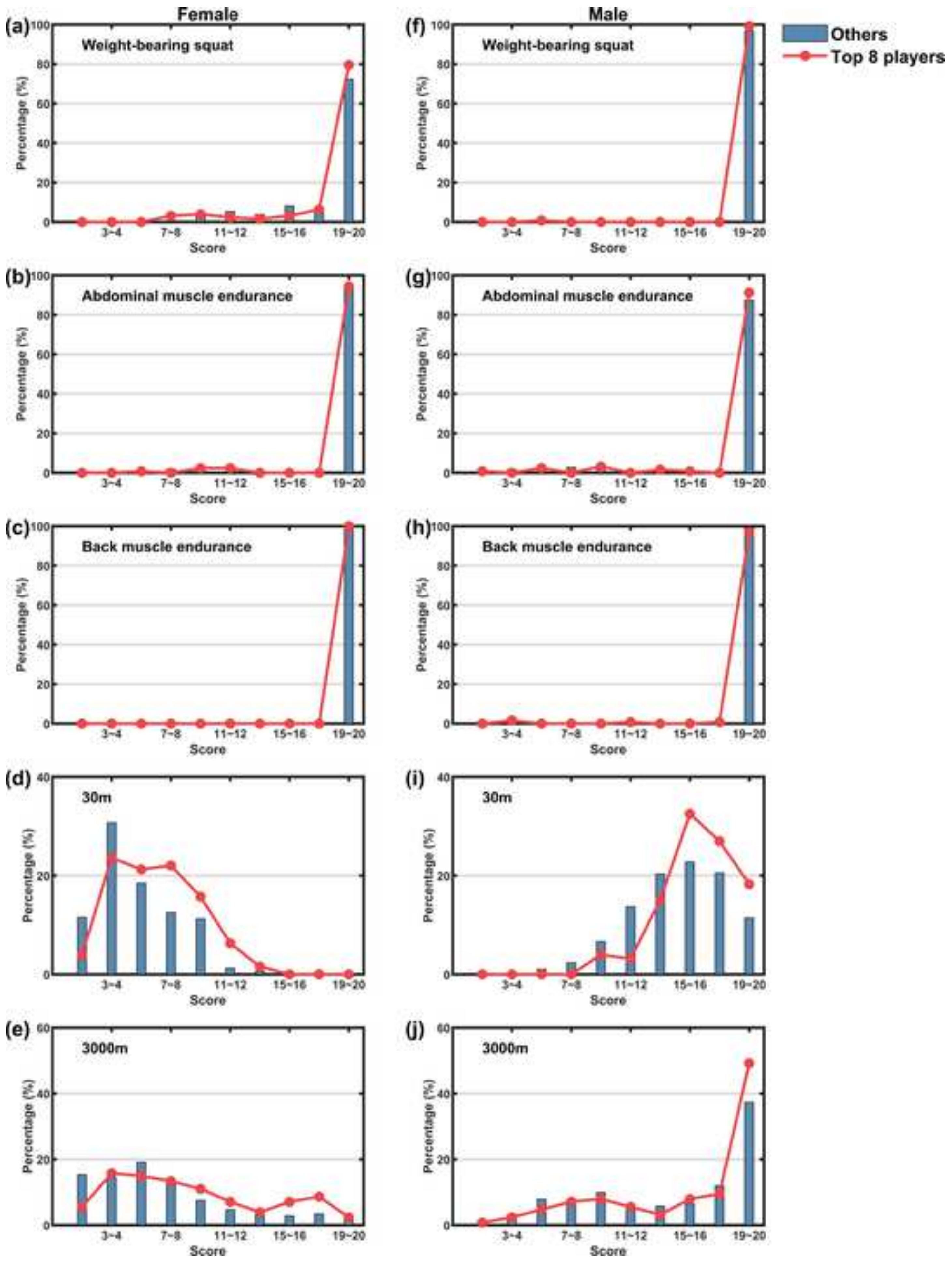

Figure5

Click here to access/download;Figure;Fig5.tif

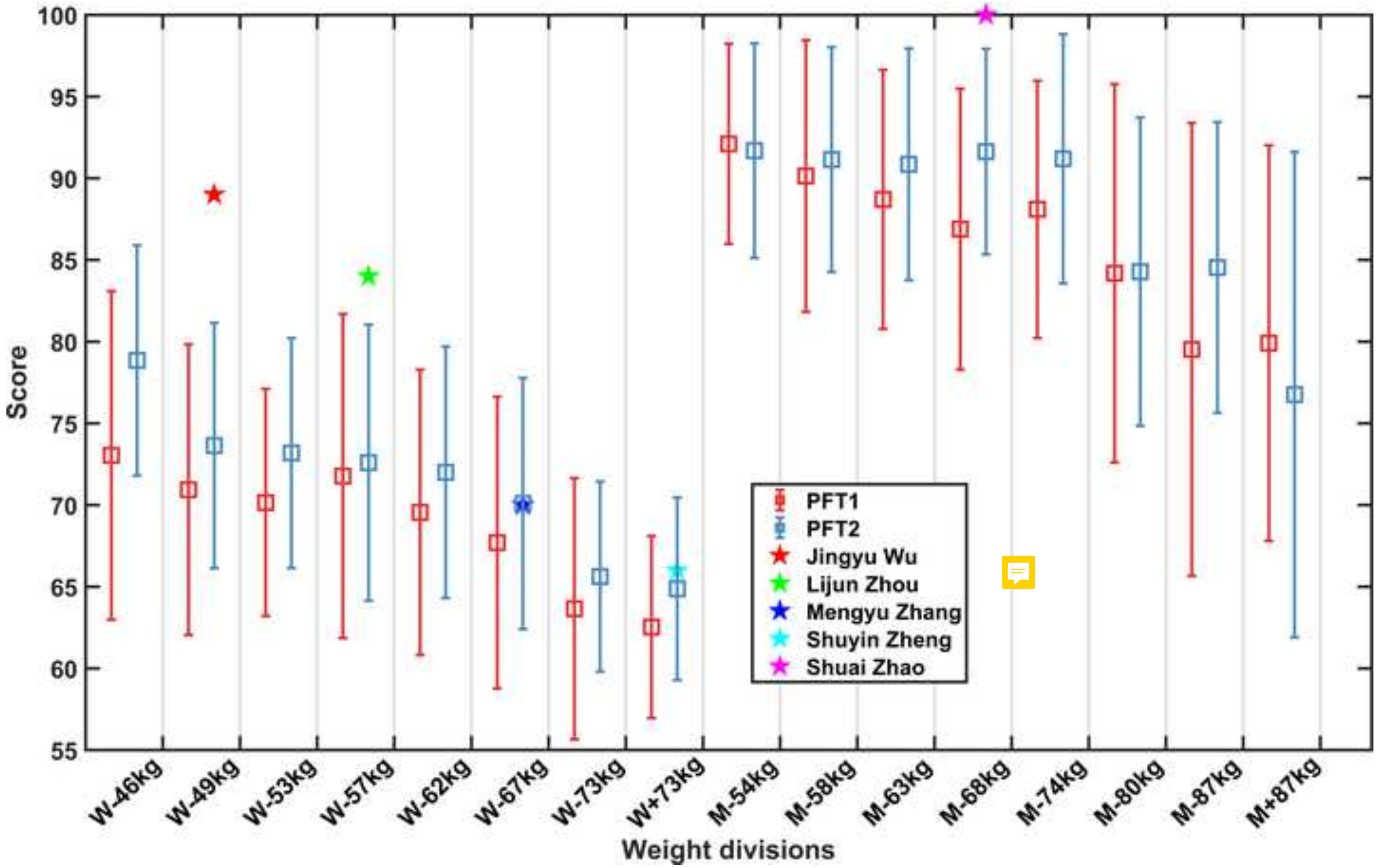

Supplement: Attachment — Submitted filename: PONE-D-21-18355_reviewer-3.pdf [file pone.0267711.s003.pdf]
