# Supplementary material for: The relationship between physical fitness and competitive performance of Taekwondo athletes
Source: PLoS One. 2022 Jun 24;17(6):e0267711. doi: 10.1371/journal.pone.0267711 (PMC9232157; doi:10.1371/journal.pone.0267711)
Supplement: Attachment — Submitted filename: response.docx [file pone.0267711.s004.docx]

List of Responses

Dear Editors and Reviewers:

Thank you for your letter and for the reviewers’ comments concerning our manuscript entitled “The relationship between physical fitness and competitive performance of Taekwondo athletes” (ID: PONE-D-21-18355). Those comments are all valuable and very helpful for revising and improving our paper, and provides guidance to us. We have studied the comments carefully and made revisions accordingly. In addition, we also upload the raw data used in our research (in supporting information), which is translated and summarised from the data in Chinese. The raw data in Chinese can be downloaded from the Chinese Taekwondo Association website (http://www.taekwondo.org.cn/). The main changes in the paper and responses to reviewers’ comments are as follows:

**Reviewer #1:** 
1) The description of the PFT test corresponds with the actual procedure of the said test, still there is no indication of any citation with scientific recognition purely devoted towards description of said test. In order to counter any doubts about the choice of PFT, appropriate citations seems more than necessary.

Response: Thanks for your suggestion, now we have added some literatures to support the PFT used in the NTCS that we invesgated in our study (Line 101-103 in the revised manuscript).

2) What kind of equipment was used to determine the outcomes of each of the 5 items in the PFT test? Please attach all of the manufacturer’s details, version/batch number, software release, etc. Even the most trivial items used in the study (i.e. jumping box, stopwatch), without which the test cannot be recreated otherwise, need to be described.

Response: The information about the equipments are listed in the Supporting information now, including the length, weight and diameter of the barbell bar, the weight of barbell, the weight of barbell clamp, the parameter of jumping box, and the model of stopwatch. However, we don’t have access to the more detailed information, such as the manufacturers of barbell and jumping box.

3) Addressing the phrases from lines 85-86, it seems only viable, to at least name the reasons (just this once) of exclusions in a bracket, i.e. (military duties, school exams, unplanned restitution, etc.).

Response: Thanks for your reminder, we have added a few more specific reasons to explain that (Line 88 in the revised manuscript).

4) After careful analysis of the attached citations, it would seem that the most troublesome aspect of the whole study is. Why did the authors decided not to include any type of a bio-monitor throughout the whole course of the research?

Most of the research teams of the cited articles, did use a wide variety of scientific equipment to precisely assess the fitness level of their subjects. This is usually done in connection with main study method (like PFT in this study) in order to prove the eligibility and accuracy of the method itself. As a side note Bio-monitors record indices such as time, speed, body temperature, physiological load, mechanical load, etc. What is more, they were allowed for use, by the World Taekwondo officials during tournaments.

Response: Thanks for your insight into this issue. Indeed, the relationship between physiological load, mechanical load and athlete performance has been studied extensively, but our aim in this paper is to investigate the relationship between the PFT test scores and the athletic performance of taekwondo athletes. To the best of our knowledge, similar PFTs are used in the German motor test, the US Army Air Forces test, the physical fitness scoring system for naval service personnel and the national student fitness test in China (Line 101-103 in the revised manuscript). These fitness tests are quicker and more efficient than biological tests and, although more crude, reflect the macro-athletic ability of the athlete. Another equally important reason is that we only have access to data from PFT tests and not biomonitoring data, although they may exist.

5) Why did the research team opt not to carry out an analysis of covariance (as “main course” or as an addendum)? As stated earlier, the amount of research participants is impressive and would suffice for said analysis. It would seem beneficial to apply covariates such as:

a. Seniority (junior/senior)

b. International acclaim

c. Years of combat experience (truly not the same as point b)

d. Day of competition/testing (as stated in the article itself, there were multiple measure periods)

e. Other factors that the research team deems worthy of adding and are aware of

Response: Thanks for your valuable comments and suggestions. Analysis of covariance is indeed a powerful tool for excluding the influence of uncontrollable and uninteresting variables on the results, however, we do not seem to be in a position to do this analysis for several reasons. The first and most important being that we do not have relevant data such as the number of years of training of the athletes, the specific days on which each athlete took the physical tests (for example, we only know that the first phase of the PFT took place on the two days of 19 September and 20 September). Regarding the several covariates you mentioned that may have an impact on the results,

a. Seniority (junior/senior): According to the rules of the NTCS, the participants of the 2020 NTCS are professional taekwondo athletes, from the provincial or municipal teams of each province, not amateur athletes. If classify them as junior and senior, they would all be senior athletes.
b. International acclaim: We have no way of knowing whether they have received international acclaim or not.
c. Years of combat experience: As we explained earlier, this data is not available to us.
d. Day of competition/testing: The physical tests and the exact dates of each competition are undisclosed (only time slots are disclosed, for example the first leg starts on 22 September and ends on 25 September). On the other hand, for the single PFT, which lasted two days, this time difference was actually considered negligible, whereas for the two PFTs, the difference in performance due to the adjustment of the athletes' training plan was much greater. As analysed in the manuscript, after the first PFT the athletes discovered that the 30 m sprint was the key to the difference in ranks, and as a result the average grade in this item improved significantly in the second PFT.

We believe that in addition to PFT results, these variables you mentioned also have an impact on athletes' performance in competition, and the lack of such data is a major limitation of this paper, so we have included that section in the final conclusions section (Line 313-316 in the revised version).

6) As far as I am concerned, the authors should specifically underline that only one tournament was monitored and continuing the narrative from remark number nr.5, challenge themselves against the knowledge from articles they have cited in this paper, how this can be seen as a limitation. Please bear in mind, that in at least of three of the citations, scientific data was gathered from more than one tournament.

Response: Thanks for your valuable comments. However, we do not consider this to be a limitation of our study, in the literatures we cited, some collected data from multiple matches (e.g., Janowski et al., 2020; Sadowski et al., 2012; Kim et al., 2019), while others indeed collected data from only one match (e.g., Maloney et al., 2018; Bridge et al., 2014; Miller et al., 2011). For the 2020 NTCS we are studying, it is a series of four competitions, each of which is complete, from the preliminaries to the final, so it’s actually not just one competition (Line 76-83 in the revised manuscript).

Reference:

1. Janowski M, Zieliński J, Ciekot-Sołtysiak M, Schneider A, Kusy K. The effect of sports rules amendments on exercise intensity during taekwondo-specific workouts. Int J Environ Res Publ Health, 2020; 17(18): 6779.
2. Sadowski J, Gierczuk D, Miller J, Cieslinski I, Buszta M. Success factors in male WTF taekwondo juniors. J Combat Sports Martial Arts. 2012;1, 47-51.
3. Kim H-B, Jung H-C, Song J-K, Chai J-H, Lee E-J. A follow-up study on the physique, body composition, physical fitness, and isokinetic strength of female collegiate Taekwondo athletes. J Exerc Rehabil. 2015;11(1): 57.
4. Maloney MA, Renshaw I, Headrick J, Martin DT, Farrow D. Taekwondo fighting in training does not simulate the affective and cognitive demands of competition: Implications for behavior and transfer. Front Psychol. 2018;9(JAN): 25.
5. Bridge CA, Ferreira Da Silva Santos J, Chaabène H, Pieter W, Franchini E. Physical and physiological profiles of taekwondo athletes[J]. Sports Medicine. 2014;44(6): 713-733.
6. Miller J, Bujak Z, Miller M. Sports result vs. general physical fitness level of junior taekwondo athletes. J Combat Sport Martial Arts. 2011;2(1): 39-44.

7) Multiple times did the authors underline the benefits of introducing the PFT testing as viable method to predict and assess competitive performance of athletes. I find the presented results minimally too broad. If possible, could the authors try to make the discussion even more interesting, by speculating how should thresholds or ranges thereof look like for different types of performance. Putting it simple, how much PFT score is necessary for qualifying to cup stage, quarter finals, semis etc.?

Response: Thank you for your suggestion that we should enrich the discussion, and thanks for the hint. In fact, it’s hard to give a clear threshold because the fitness level of the players varies from weight categories. We have discussed a similar issue as much as possible (section 3.1.2), the results illustrate that there is a significantly negative correlation between the PFT ranking percentage and its proportion in the top 8 athletes, for example, an athlete with a PFT ranking of 10% or less has a 17% chance of reaching the top eight in the competition, while an athlete with a PFT ranking of 70%-80% has only about a 6% chance of reaching the top eight (as shown in Fig 3).

**Reviewer #2:**

Introduction

- line 39 I’m not sure what is meant by “increase in the antagonism between two competitors”. The whole sentence could be deleted.

Response: This statement was indeed somewhat subjective and unsupported by the literature, and we have removed it at your suggestion.

- line 40 “previous rules” maybe giving a year when they were introduce will guide the reader which rules were meant

Response: Thanks for your insight into this, we have modified it to “pre-2015 rules” (Line 39 in the revised manuscript).

Methods

- line 77 chart of a timeline would increase the readability of the process. What is the first and second race? Do you mean the subsequent matches leading to the finals?
- Did the same athletes take part in consequent races or is that organised for different groups of athletes? The number of participants suggested the second way but it was not described clearly.
- Were the PFT results taken into account to be included in the further stage of competition or it didn’t have an influence on the result of the competition? It is crucial to understand how the both scores relate with each other.

Response: We apologise for the unclear and misleading statement about the process. We have reorganised this part of the description (Section 2. Materials and methods).

Specifically, the 2020 NTCS contains four competitions and used a scoring system, (Line 76 in the revised manuscript), all the athletes were required to compete in all four competitions (Line 86-87). After each competition athletes were awarded points according to their competition ranking and the total number of points from the four competitions determines the final ranking of the athletes in the 2020 NTCS.

According to 2020 NTCS rules, athletes who score below 50 in the PFT are directly disqualified from the competition. After the top 8 players are determined in each competition in each competition, the top four of these eight athletes in terms of PFT ranking go directly to the semi-finals, while the last 4 players are regarded as tied for fifth.

- line 80 change “fourth races” to “fourth race”
- line 82 I believe that authors refer to Taekwondo Competition Rules, not PARA Taekwondo Competition Rules. It needs to be explained or changed.

Response: Thanks for your insight into this, we have modified these accordingly.

- line 84 what is meant by “professional” - does it mean that the competition was closed to amatours? If that’s true, adding selection criteria for competition would help to understand the selection process.
- Other participants' characteristics are missing. No information re the age range. Is the National Taekwondo Championship Series for seniors only or junior athletes are also competing?

Response: Yes, it means the 2020 NTCS was closed to amatours, and all the athletes were senior athletes from provincial teams in all provinces of China (Line 85-86). Unfortunately, however, the organising committee does not disclose data on the age of these athletes, their years of training and whether they have won international acclaim. This is a limitation of our study and we have added this part of discription in the Line 313-316.

- In the results the differentiation between the national team was addressed - it should be described in the methods section.

Response: Thanks for your advice, it is now added in the Line 124-128.

- line 96 Table 1. How has the grading standard developed? Any references? Why is only one of the PFT grading standards different for female and male athletes?

Response: The PFT items, scoring criteria and test criteria are all decided by the NTCS event organising committee, including the Chinese Taekwondo Association and the Jiangsu Provincial Sports Bureau. (We have added this sentence to the revised manuscript, in Line 122-124). For the scoring criteria used in the 2020 NTCS PFT, we found no published literature to prove its reliability. On the other hand, as we used the rankings of the athletes' fitness tests in our analysis, we avoided, as far as possible, the bias caused by possible uncertainties in the scoring system.

- line 99 word “produced” should be changed to “determined” or similar

Response: This word has been replaced.

- Ethics statement should be provided for this study, although authors state that it is not required. It is Human Subject Research involving human participants.

Response: We have added the Ethics statement in Line 331-335.

- Statistical methods should be described. Why and which analysis were conducted? No explanation can be found in this section and it’s difficult to find the clarity in the results descriptions.

Response: The data statistics used in this paper include each athlete's PFT score, percentage of PFT ranking, competition placements and points, and these are now descriped in the Section 2 in the revised manuscript.

Results

- This part of the manuscript is divided into sections - the same structure could be adapted to describe statistical analysis in the Methods section.

Response: Thanks for your advice, however, we think this part could be kept because the description of this part of the statistics is not sufficient to support a subsection.

- line 107 Total points calculation should be moved to the methods section

Response: Thanks for your valuable comment, we have moved this part to the section 2.

- line 133-136 The title and description of the figure should be separated.

Response: Thanks for your suggestion. We have revised them accordingly.

- line 149 “quarterfinals” how this term relates to the term race? The descriptions in the methods section should clearly explain that.

Response: Athletes entering the quarter-finals means entering the top eight, and in 2020 NTCS means they can get points. In addition, from the quarterfinals to the semifinals, the top 4 of these 8 athletes are directly selected through the PFT rankings. Therefore, in order to avoid the bias caused by this competition system to study the influence of PFT results on athletes' competitive performance, we discussed entering the quarterfinals (section 3.1.2) and winning the first place in the quarterfinals (section 3.1.3) separately.

- line 183 “eight heavyweights'' should be changed into “weight categories” or “weight divisions”. Although standardEnglish is used, some terms in the text are misleading.

Response: Thanks for your advice, all the “heavyweight” are replaced by “weight category” in the revised manuscript.

- line 253 “quite good” should be changed to different, more objective term

Response: This statement has been revised.

- line 255 and Fig 5 names of participants were presented - did the researchers receive the consent to use the personal details? Prior to sharing human research participant data, authors should consult with an ethics committee to ensure data are shared in accordance with participant consent and all applicable local laws. Data sharing should never compromise participant privacy. It is therefore not appropriate to publicly share personally identifiable data on human research participants.

Response: Thanks for your kindly reminder, we have removed all the real names of the five elite athletes that appeared in the original manuscript.

- Knowing the rationale for the inclusion of each analysis and having a clear description of which participants, from which stage of competition took part in it, would have increased the understanding of the whole research design. I believe that each analysis was conducted with the purpose but neither methodological section nor the discussion explains their value and intention in an intelligible fashion.

Response: Thanks for your valuable comments. The main aim of this paper is to study the relationship between PFT results and taekwondo athletes' competitive performance by analyzing the results of four competitions in the 2020 NTCS. In order to study this issue, we first discussed the relationship between the athletes’ total scores in four competitions and their PFT results, and found that there is an exponential positive correlation between the total scores and PFT rankings (Section 3.1.1); we conducted research on the relationship between entering the quarterfinals (section 3.1.2) and winning first place in the semi-finals (section 3.1.3) and their PFTs, respectively, and found that athletes with relatively good PFT rankings have a better chance of achieving good results in the competition; then we carried out the individual PFT results of the athletes, analysis shows that 30-meter running is a key physical fitness index that determines the athlete's competition performance (section 3.2); finally we discussed the athletes' PFTs further in section 3.3.

Conclusions

- Part of this section should be added to the discussion part of the manuscript.
- Had the research questions been clearly and specifically formulated in the introduction or methods, the conclusions could have been more precise.

Response: Thanks for your valuable comments, we have enriched the discussion in the revised manuscript. As the last paragraph of the introduction declares, the question of our study is to figure out the relationship between PFT results and taekwondo athletes' competitive performance.

**Reviewer #3:**

- In summary, you need to used proper terminology with regards to Taekwondo. For example we do not use “race” in TKD but “match” or competition. there are other vocabulary that I highlighted for you.

Response: Thank you for pointing this out. We have addressed all the “race” in the manuscript into “competition”.

- You need to include the studies on the validity and reliability of the Physical Fitness Tests (PFT) used in this study. Who devised The scoring system used in your study and was it tested and reliable?
- Line 87, you need to explain each component of PFT separately with reference and validity and reliability scores.
- Line 96, what is the reference for this scoring?

Response: The PFT items, scoring criteria and test criteria are all decided by the NTCS event organising committee, including the Chinese Taekwondo Association and the Jiangsu Provincial Sports Bureau. (We have added this sentence to the revised manuscript, in Line 122-124). For the scoring criteria used in the 2020 NTCS PFT, we found no published literature to prove its reliability. On the other hand, as we used the rankings of the athletes' fitness tests in our analysis, we avoided, as far as possible, the bias caused by possible uncertainties in the scoring system. And the item set-up in 2020 NTFS PFT is similar to the German motor test, the US Army Air Forces PFT, the physical fitness scoring system for naval service personnel and the national student fitness test in China (Line 101-103 in the revised manuscript).

As Fig. 4 shows, the scale does not seem to clearly differentiate between the athletes' weighted squat, back endurance and abdominal endurance abilities (almost all athletes achieved a perfect score). This could be a potential source of bias in the results as there are two explanations, one being that the professional athletes did have good leg strength, abdominal endurance and back endurance, and the other being that the standard for a perfect score was not high enough. We have added this part of the discussion to the revised manuscript (Line 316-319).

- Your tables are very confusing and need to be redone. You have used too many tables and figures. you need to decrease the numbers and do not use table and figure to state the same data.

Response: Thanks for your suggestion, but we believe that every figure and every table in the manuscript is essential to our study. Firstly, Tables 1 and 2 give the scoring rule for the 2020 NTCS and scoring criteria for the PFT, respectively, which are the basis for the results that follow; Figure 1 shows the relationship between total points and PFT results; Tables 3 and 4 list the parameters and goodness of fit of the 18 fitted curves in Figure 1, which we believe are necessary for the analysis of the results; Figure 2 shows the PFT rankings of the top eight male athletes and top eight female athletes in the four competitions; Figure 3 summarises the probability of athletes with different PFT ranking percentages reaching the Top 8 in all four competitions, which is a further insight into Figure 2; Table 5 lists the number of times athletes with different rankings won the first place after reaching the Top 4, which is also beneficial for studying the relationship between competition performance and fitness of taekwondo athletes; Figure 4 shows the difference between the individual physical test scores of athletes who reached the Final 8 and those who did not; Figure 5 compares the PFT scores of the elite national team athletes with the average PFT scores of the athletes in their weight categories, again showing the importance of physical fitness to competitive performance.

We think no two figures or tables state the same data.

- Your discussion portion of your paper lacks the comparison with other studies and stating the reasons behind your finding in light of the literature. This is very crucial and essential for the success of a paper.

Response: Yes we totally agree with you that the comparison with other studies is very crucial for a research paper, actually we have worked hard to do this, for example, in Line 233, Line 257 and Line 305. However, as there are not many relevant studies, more comparisons seem very challenging.

- One last but most concerting is the ethical issues of this study. It is unethical to include the name of athletes or any identifying data about the athlete in a scientific peer reviewed paper

Response: Thanks for your kindly reminder, we have removed the names of any athletes involved in the paper.

- Line 99, was this base on PFT scores or TKD competition?
- Line 101, why? This could be a source of bias.

Response: This is based on PFT scores. Because according to the rule of 2020 NTCS, after the top 8 players are determined, the top 4 of these 8 athletes in term of PFT ranking will go directly to semi-finals, but not based on TKD competition. We acknowledge that this system may create some bias, mainly in that it is possible that athletes who do not do well in the PFT also have the opportunity to advance in the 8-in-4. This bias may be responsible for the exponential, rather than linear, relationship illustrated in Figure 1 when discussing the relationship between the final total score and PFT result (discussed in Line 188-192). Therefore, in order to avoid this bias, the relationship between " Entering the quarterfinals" (section 3.1.2) and " Winning in semifinals" (section 3.1.3) and the PFT results are analysed and discussed separately.

- Line 110, is this your idea or has it been published and validated?

Response: The organising committee of the 2020 NTCS gave the rules for points, as shown in Table 1, with 100 points for first place, 60 points for second place, 36 points for third place and 21.6 points for fourth place. When we trace the exponential relationship between total points and the PFT results (section 3.1.1), we found that the scoring rule of 2020 NTCS can also be expressed exactly as an exponential function. We have therefore summarised the scoring rule of 2020 NTCS as a exponential function, which can be easier for other tournaments to refer to and generalise, and it is easy to verify this formula.

- Line 187, this s very confusing. Was there only one competition? Are you referring to each match and each race? You need to clarify by winning the competition do you mean the athlete got gold medal?
- Line 189, this is very confusing which one is the athlete's rank in PFT and which one is their rank in the competition?

Response: Perhaps the introduction of the 2020 NTCS was not presented clearly enough before, which led to the confusion here. We have now revised the writting of the 2020 NTCS competition rules and PFT rules in the methods section and hope to make the results and discussion in this section clearer.

As the 8-in-4 is directly determined by the PFT results and not the competition results (as discussed earlier, there may be some bias here), in order to examine the relationship between athletes' competition performance and fitness levels, we need to devide the discussion into two parts, one is that presented in section 3.1.2, the other one is “whether athletes with better PFT results have a higher probability of winning the first place after reaching the semifinal” as discussed in this section.

There are 4 competitions in 2020 NTCS (first column in Table 5), in the case of the male athelets, for example, each competition produced eight players who were first place, as there were eight weight categories. For these 8 first place athletes, it is helpful to count their PFT ranking among the top four athletes in their respective weight categories. Assuming there is no relationship between competition performance and fitness, the number of times the No. 1, No. 2, No. 3 and No. 4 ranked athletes in the PFT have won first place in a competition should all be 2, however, actually it is 4, 1, 2, and 1 (as the first line of number in Table 5), which means that the athlete who ranks first in the physical test among these four is more likely to win first place in the competition.

- Line 218, were these differences statistically significant? Please state.

Response: This phrase was considered too colloquial and also redundant, so we deleted it.

- Line 236, how are these compared to other sports and other references. In your discussion you need to compare your results to other papers and state the reasons.

Response: The comparisons we make here are between the athletes studied in this paper, as the exact same fitness test criteria are not used in the other literature, we are unable to make comparisons with other sports and other references. In fact this conclusion can already be drawn from Figure 5.

- Line 254, higher than who and what is you reference?

Response: Thanks for your comment, we have reorganized this sentence, now it’s in the Line 286-290 in the revised manuscipt.
