# Supplementary material for: The relationship between physical fitness and competitive performance of Taekwondo athletes
Source: PLoS One. 2022 Jun 24;17(6):e0267711. doi: 10.1371/journal.pone.0267711 (PMC9232157; doi:10.1371/journal.pone.0267711)
Supplement: Attachment — Submitted filename: Response to Reviewers.docx [file pone.0267711.s005.docx]

**Responses to the Editor and Reviewers**

Dear Editor Durkalec-Michalski,

Thanks for your valuable comments. In the following we answer each specific point (in blue).

Descriptions of figures are unclear and it is difficult for the reader to fully accurately interpret the presented data - the quality of figure descriptions and their descriptions under the figures should be clearly improved.

Thanks for your insight into this. We have improved the description of the figures, both in the text and in the legend, especially the descriptions of Figs 1 to 4 have been significantly modified. The missing legend has been added to Fig 2 (red ladders). We hope these modifications could make the figures easier to understand.

Lines 173-174 - Remove this torch "high-flown" stylistically sentence (“Potential possible causes for this phenomenon will be discussed later”). At the same time, make it clear in the discussion that you are referring to these observations from the results.

Sorry for the colloquial expressions, this sentence has been removed. And we have made it clear that the discussion was based on the results in the previous section, for example, in Line 303 of the revised manuscript we stated that "This may explain the results in section 3.1.1."

Consider adding legends under the tables to help the reader understand the tables.

Thanks for the valuable suggestion, we have now added some legends to make the table easier to understand, e.g., Table 3 and Table 5.

Tables should be properly edited to make it easier to follow (breaking descriptions into lines makes it difficult.

We have rearranged Tables 3 and 4, which may not be easy to follow as mentioned by the editor.

The name of table 4 (description) is unreliable. The table should be fully and properly named so as not to raise doubts and not to send the reader to look for elsewhere.

This too brief description has now been reformulated to "Table 4. The fitting coefficient (a and b), the Goodness of Fit (R^2^) and the number of athletes (N) in Fig. 1d-e. "

Statements such as "This feature is more obvious for" should be limited - statements "obvious" are not entirely accurate in the discussed contexts.

Thank you for pointing this out, we have replaced the word "obvious" to "pronounced" to make it more academic.

Again - the description in tables, figures and in the text must allow for an authoritative and unambiguous assessment of what they refer to and what they indicate. At the moment, only the reader who has read the work very carefully, are they clear. It is necessary to precisely and clearly describe what and what these figures indicate, with whom and when.

We agree with the editor that the description of the figures and tables should be clear and easy to understand, and we have modified almost every figure and table description in the text accordingly for this purpose.

Lines 256-257 – “…athletes are pretty good.” – reword this using more scientific language and past time. (Line 239?)

Thanks for the advice, we have now reformulated this sentence to read "This means that … of these professional Taekwondo athletes all achieved the standard of excellence set by the General Administration of Sport of China."

Line 258 – avoid using abbreviations “didn’t”. (Line 240?)

This abbreviation has been revised.

Consider whether in every context in the work naming athletes who train taekwondo as "players" is reliable - I have some concerns here.

We agree with the editor that "players" may be not a appropriate word here, we have replaced this word with "athletes" in the revised manuscript.

The discussion is quite poor when it comes to comparisons with other studies.

In this revised version, we have made significant modifications in the section 3 (Results and discussion) according to the editor and the reviewer’s suggestions, which, we hope, has improved the disussion and made it more clear. Some major modifications, including but not limited to Lines 159-162, Lines 208-211, Lines 272-280 and Lines 286-288.

A large part of the conclusions and limitations is basically a discussion - please move it to the previous section. Limit your conclusions to what has actually been shown.

Thanks for pointing out this, we agree with the editor that that some sentences should be placed in the discussion section, specifically, we have moved Lines 313-316 in the previous manuscript to Lines 208-211 in the revised version, moved Lines 316-319 in the previous manuscript to Lines 286-288 in the revised version. Some other modifications in the conclusions could also be clear seen in the tracked version of manuscript.

Reviewer #1: Starting off with a appraise, the Authors managed to address most of the issues pinpointed by the reviewers, in an orderly and clear manner. Necessary addendums as well as removals have substantially enriched the text as a whole. As stated previously, some of the less fortunate terms were present, but the Authors managed to correct them this time.

Thanks for your overall comments. In the following we answer each specific point (in blue).

Without further ado, I shall voice out the most important aspects of the current state of the article:

1) The Description of the PFT test…

From where I am standing, the addendums in lines 101-103 clear out any doubts.

Thank you, further modifications have also been made in this revision, for example, in Lines 105-109, Lines 110-111 and Lines 125-126 in the revised manuscript.

2) What kind of equipment was used…

Necessary corrections have been applied. It is perfectly understandable, that some of the more “mundane” and “less spectacular” items may have been manufactured so long ago, that the detailed information may have been next to impossible to obtain.

Thank you for your understanding, we agree with the reviewer that these informations are important for other researchers.

3) Addressing the phrases from lines 85-86…

The abovementioned issue has been addressed accordingly.

4) After careful analysis of the attached citations…

The Authors did underline the separation from biomonitoring towards a more macro-athletic approach. The limitations were provided in the renewed version of the manuscript.

Thanks for your comment, although this more macro-athletic approach has its limitations, we think it is still meaningful as it is at least a complement to the bioassay approach.

5) Why did the research team opt not to carry out an analysis…

The most hampering limitations were added by the research team in lines 313-316. The provided reasons are understandable and I strongly encourage taking them into consideration, which may finally result in obtaining data viable for an analysis of covariance. Maybe it was not aired specifically enough – no World/Asian/Olympic/Cup medalists whatsoever in said group? Just a general quota would suffice – i.e. 11 gold/22 silver/33 bronze etc.

Thanks for the reviewer’s suggestions, and we understand the concerns of the reviewer. We are sorry that we are not in a position to adopt this recommendation as we failed to obtain these additional information. However, we believe that these additional information, although beneficial for doing an analysis of covariance, will not affect the main findings of this study. Athletes' competitive performance is certainly also related to other factors such as taekwondo skills, mental fitness and competition experience (which would be related to whether or not they have previously won honours), but as our study only discusses the relationship between athletes' physical test scores and their competitive performance, the current data and statistics should be sufficient to justify the conclusions of this paper.

6) As far as I am concerned, the Authors should specifically underline…

The appropriate lines and addendums were administered by the Authors.

7) Multiple times did the Authors underline the benefits of introducing the PFT testing…

In the light of multiple alterations carried out in the text, my initial doubts have been dispelled. The percentages presented in Figure nr.2 are clear. Still, I strongly suggest tackling the issue in future research regarding thresholds in the PFT test.

Thanks for you valuable suggestion, some of the limitations in this study will be discussed and improved upon in more detail in subsequent studies.

Reviewer #3: Thank you for your revisions. It reads much better.

Thank you for your contribution to the improvement of this manuscript. In the following we answer each specific point (in blue).

However, you have not provided any evidence/references on the individual tests of the PFT reliability, validity or any reference on the way they were performed in your study or the normal values of each test in comparison to TKD athletes in your study. You have not explain the utility of each test and significance of each test physiologically and why they should be included in the battery of the tests.

Thanks for your valuable comments and sorry for the limited description of the physical fitness test in previous manuscripts. We have added the meanings of this battery of physical tests to the text, which reads "This battery of tests can be considered as an indicator stand for various aspects of an athlete's athletic ability, including agility, explosive power, strength, aerobic capacity and anaerobic capacity, which has been viewed as a multidimensional structure that reflects motor performance ability [27]", and indicated the utility and physiological significance of each individual test, for example, "Weight-bearing squat is used to test the strength of the quadriceps, gluteus maximus and other lower limb muscles [28]. ", "The 30-meter sprint test is designed to measure speed ability [23]", "The 3000 m run is a test of aerobic endurance [23]", whereas abdominal and back endurance are measured in a similar way to [29] (but not exactly the same), the specific details of each individual test are set out in the revised manuscript (Lines 110-133).

Unfortunately no reference was found to demonstrate what normal values should be achieved by TKD athletes in these individual tests. However, we believe that since the subject of our study in this paper is to compare the relationship between competitional performance and physical test scores among these professional TKD athletes who participated the 2020 NTCS, and not to compare them with other athletes, the absence of the "normal values" seems does not affect the conclusions.

You also have not provided any p values for the differences you mentioned in various athlete groups, male versus female to show statistical significance.

Sorry the unclear writing, we have tried to clarify this in the previous manuscript in Lines 142-143, which reads "All p-values in this article are less than 0.001, and will not be marked hereafter", however, it seems not clear enough especially as the reader tends to forget this when reading later. We have now removed the Lines 142-143 in the previous version of manuscript and instead, we have clarified p-values where they occur. For example, in Line 204 and Line 299.
