# Supplementary material for: The relationship between physical fitness and competitive performance of Taekwondo athletes
Source: PLoS One. 2022 Jun 24;17(6):e0267711. doi: 10.1371/journal.pone.0267711 (PMC9232157; doi:10.1371/journal.pone.0267711)
Supplement: Attachment — Submitted filename: Response to Reviewers.docx [file pone.0267711.s006.docx]

**Responses to the reviewers**

Reviewers' comments to the Author:

Reviewer #3: Thank you for the revisions. Allmy concerns have been addressed. However, there some grammatical and other minor errors that require revisions as follow:

Thanks for your careful checking. We have corrected all the following typos and grammatical errors the reviewer raised.

line 25: specifically in increase of explosive power. pllease change to "specially enhancing the explosive power."

Modified.

Line 57, "the PFTs performed are all conducted". change to: "the PFTs performed were all conducted"

Corrected.

Line 61, "Aware of the important role"change to "Being aware of..."

Corrected.

Line 76, "The 2020 NTCS contains four competitions, two PFTs and used a scoring system.", change to, "The 2020 NTCS contained four competitions, two PFTs and a scoring system."

Modified.

Line 116, "endurance of the abdominal muscles, the test subject lies prone on a jump box with the"

Should be, "endurance of the abdominal muscles, the test subject lies supine on a jump box with the"

Corrected.

Line 120-121, "back muscle endurance is the same as abdominal muscle endurance, except that the athlete lies on the jumping box" should be:"back muscle endurance is the same as abdominal muscle endurance, except that the athlete lies prone on the jumping box"

Corrected.
